# Supplementary figures and images for: Covalent disruptor of YAP-TEAD association suppresses defective Hippo signaling
Source: eLife. 2022 Oct 27;11:e78810. doi: 10.7554/eLife.78810 (PMC9728995; doi:10.7554/eLife.78810)

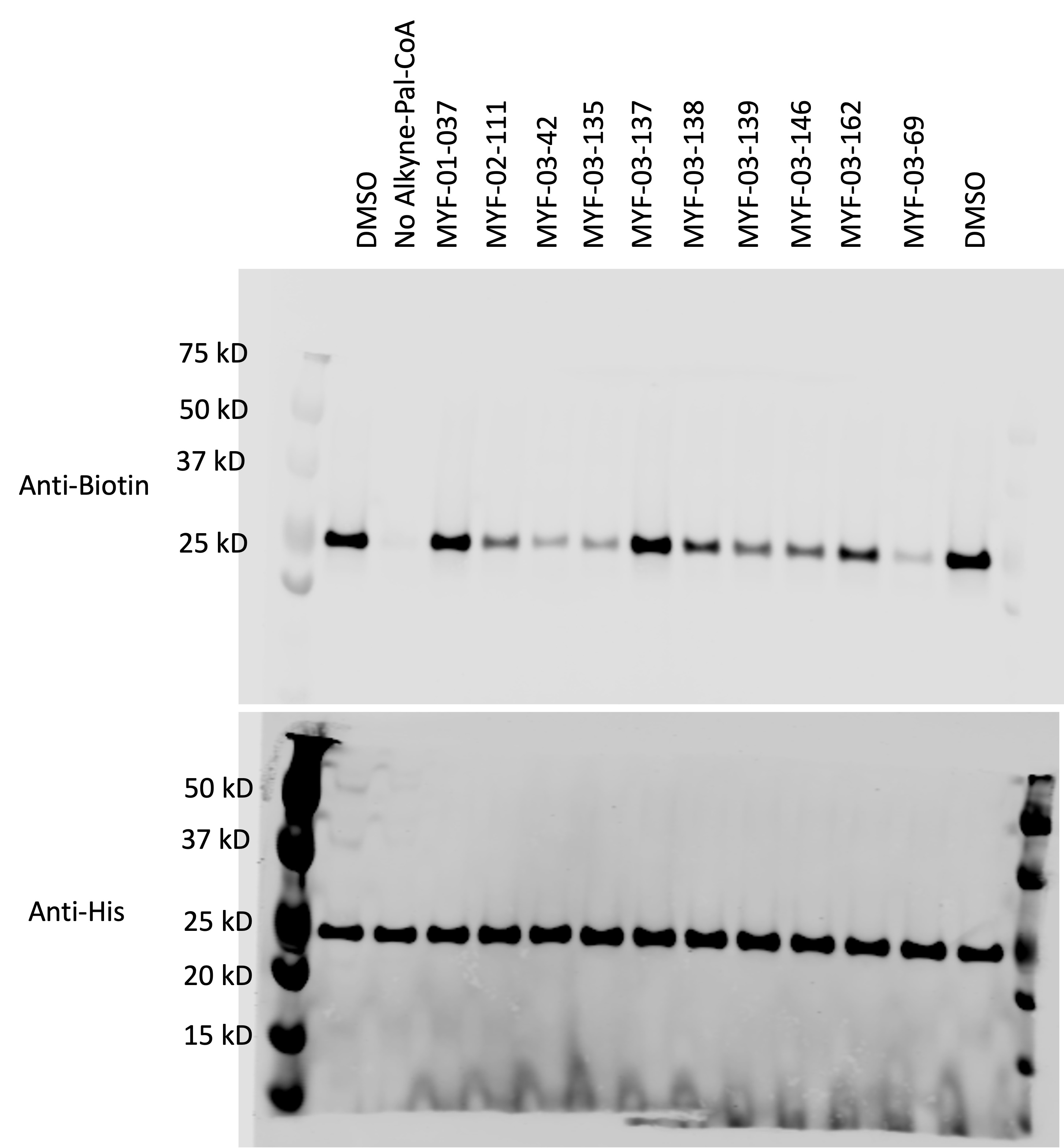

Supplement: Figure 1—source data 1. [file elife-78810-fig1-data1.zip › Figure 1-figure supplement 3-source data 2.jpg]

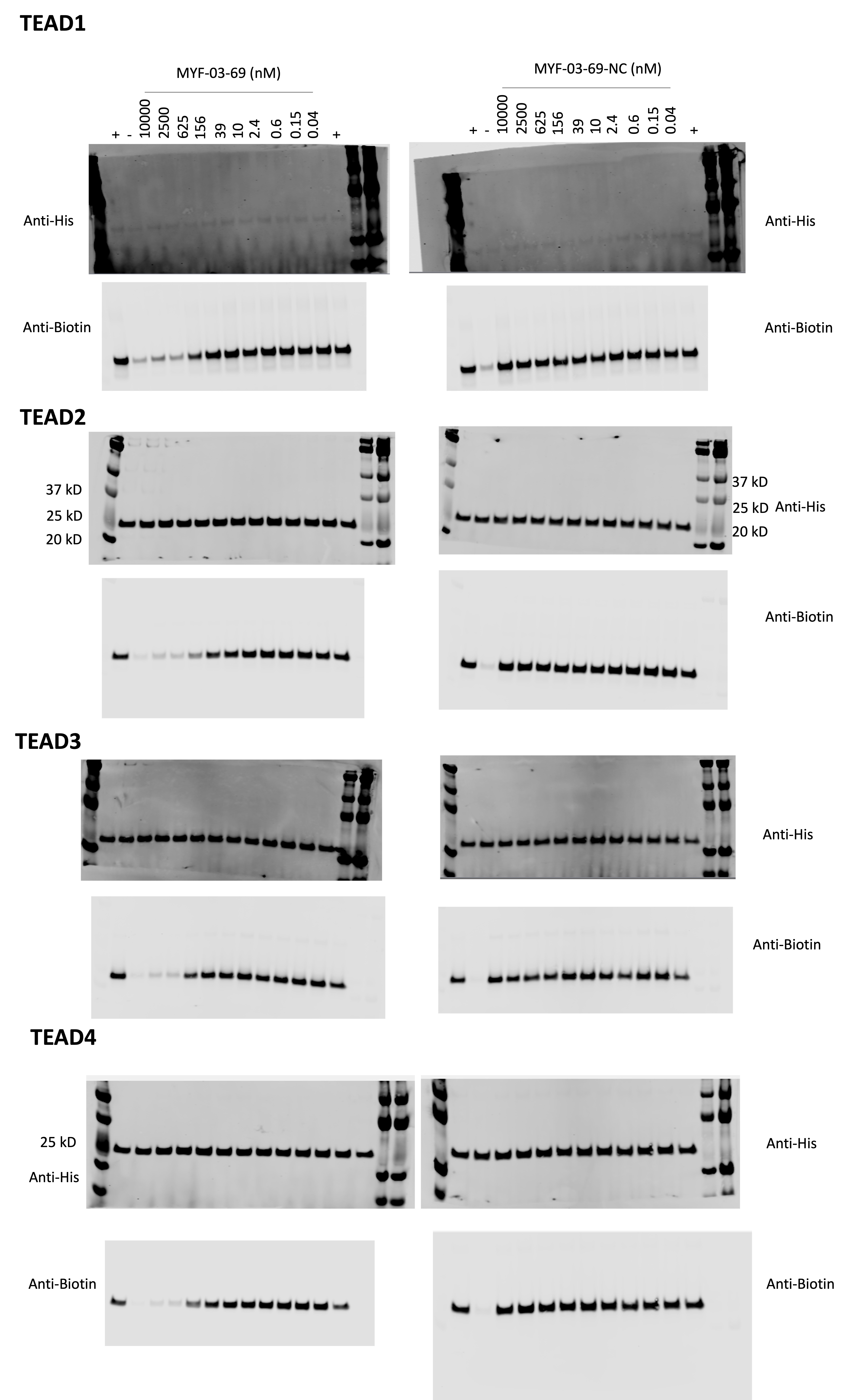

Supplement: Figure 2—source data 1. [file elife-78810-fig2-data1.zip › Figure 2-figure supplement 4-source data 1.jpg]

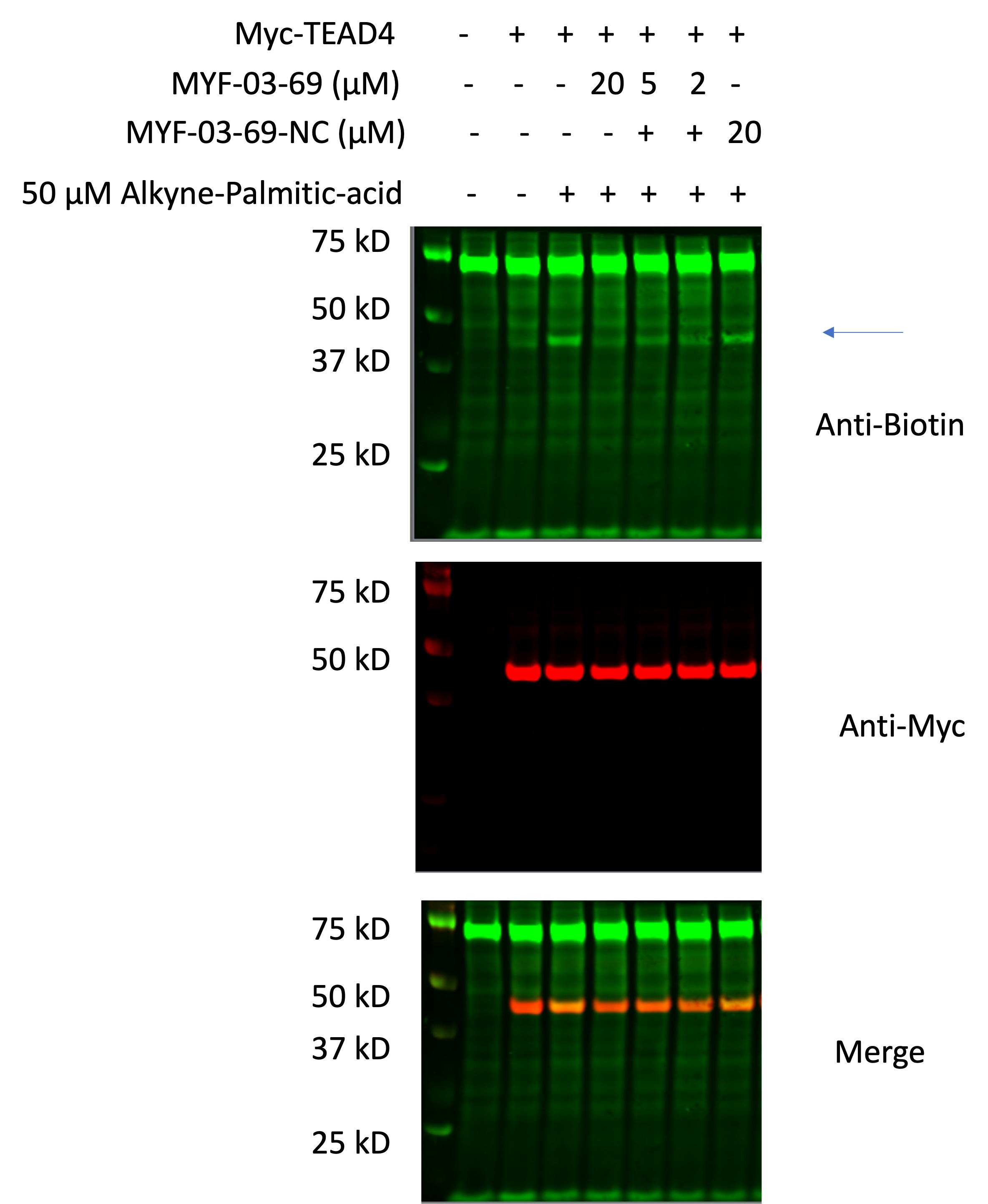

Supplement: Figure 3—source data 1. [file elife-78810-fig3-data1.zip › Figure 3-source data 2.jpg]

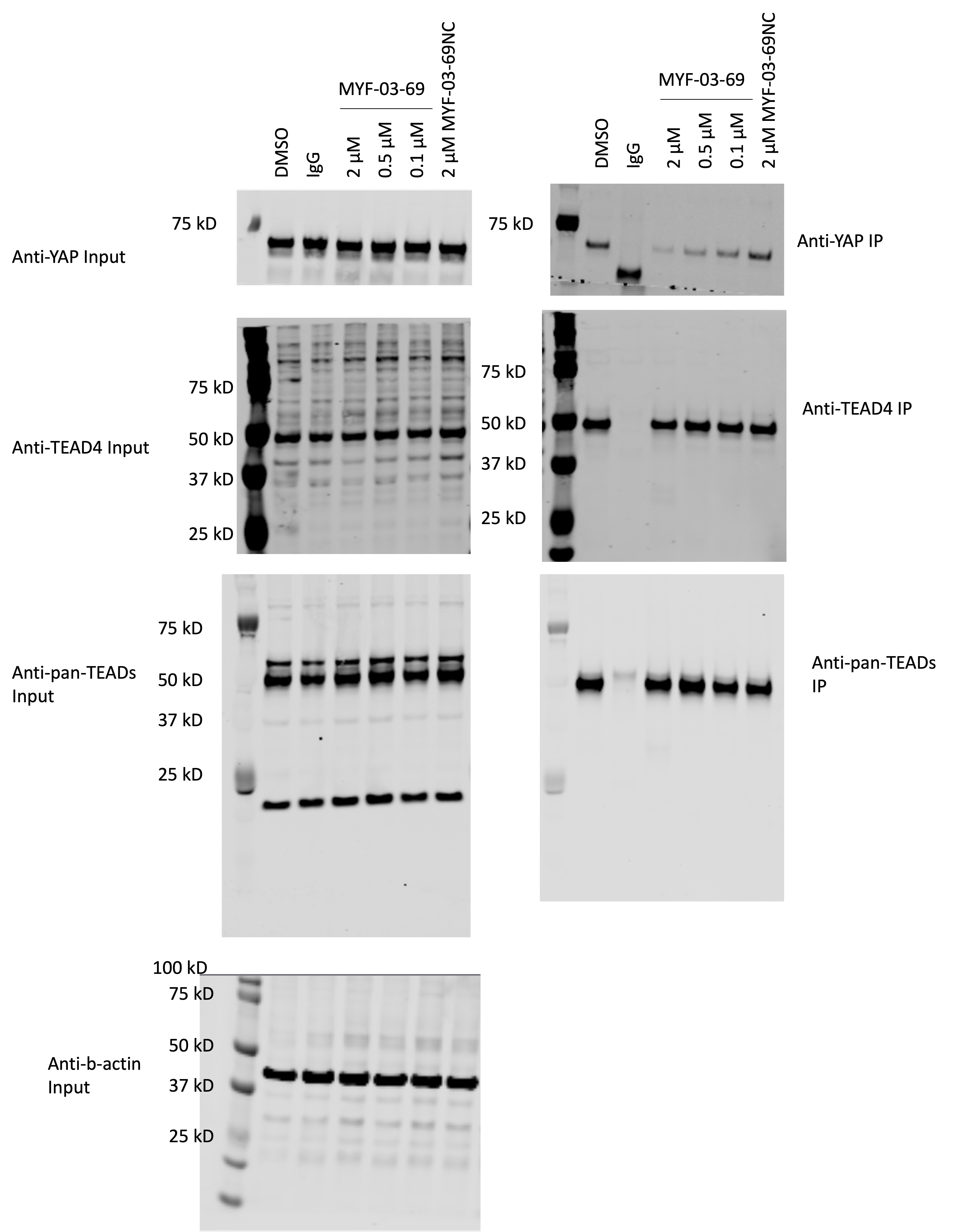

Supplement: Figure 3—source data 1. [file elife-78810-fig3-data1.zip › Figure 3-source data 4.jpg]

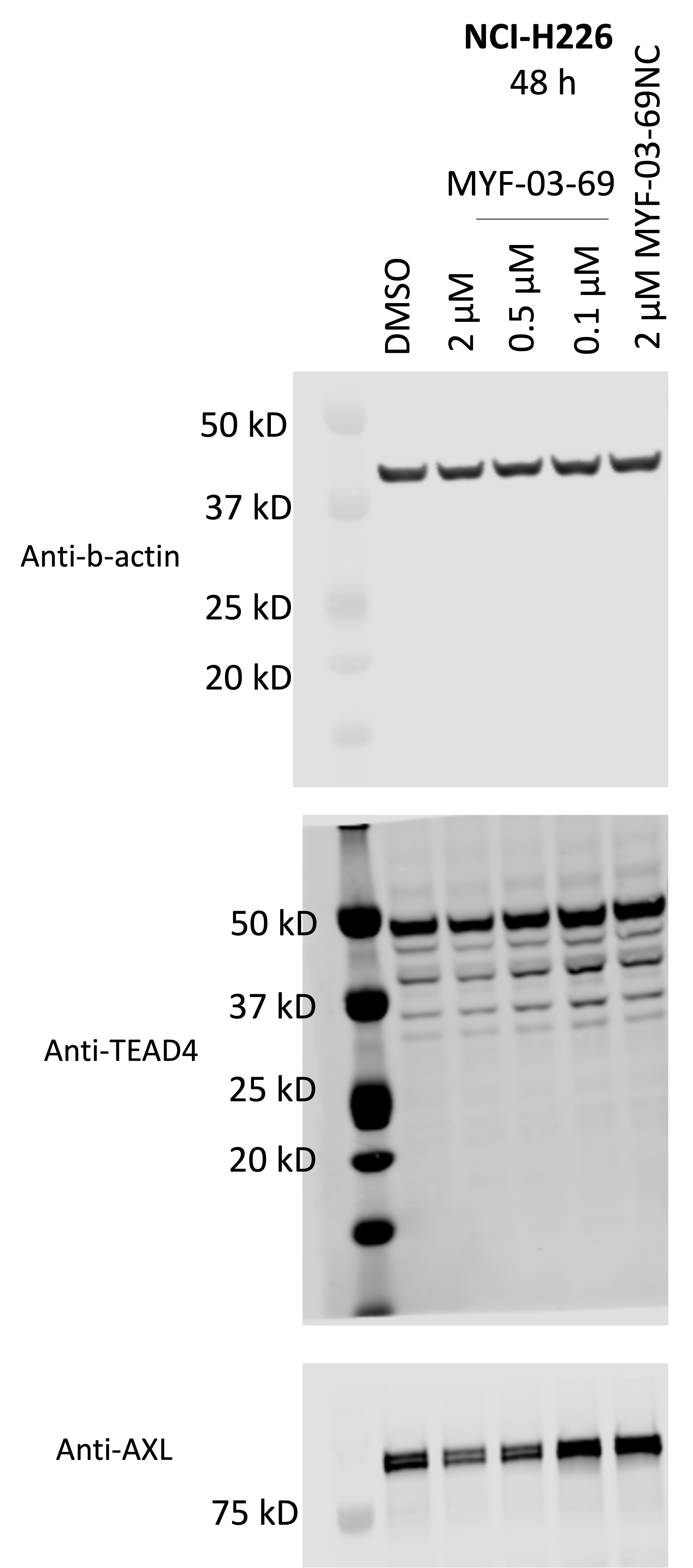

Supplement: Figure 4—source data 1. [file elife-78810-fig4-data1.zip › Figure 4-figure supplement 7-source data 2.jpg]

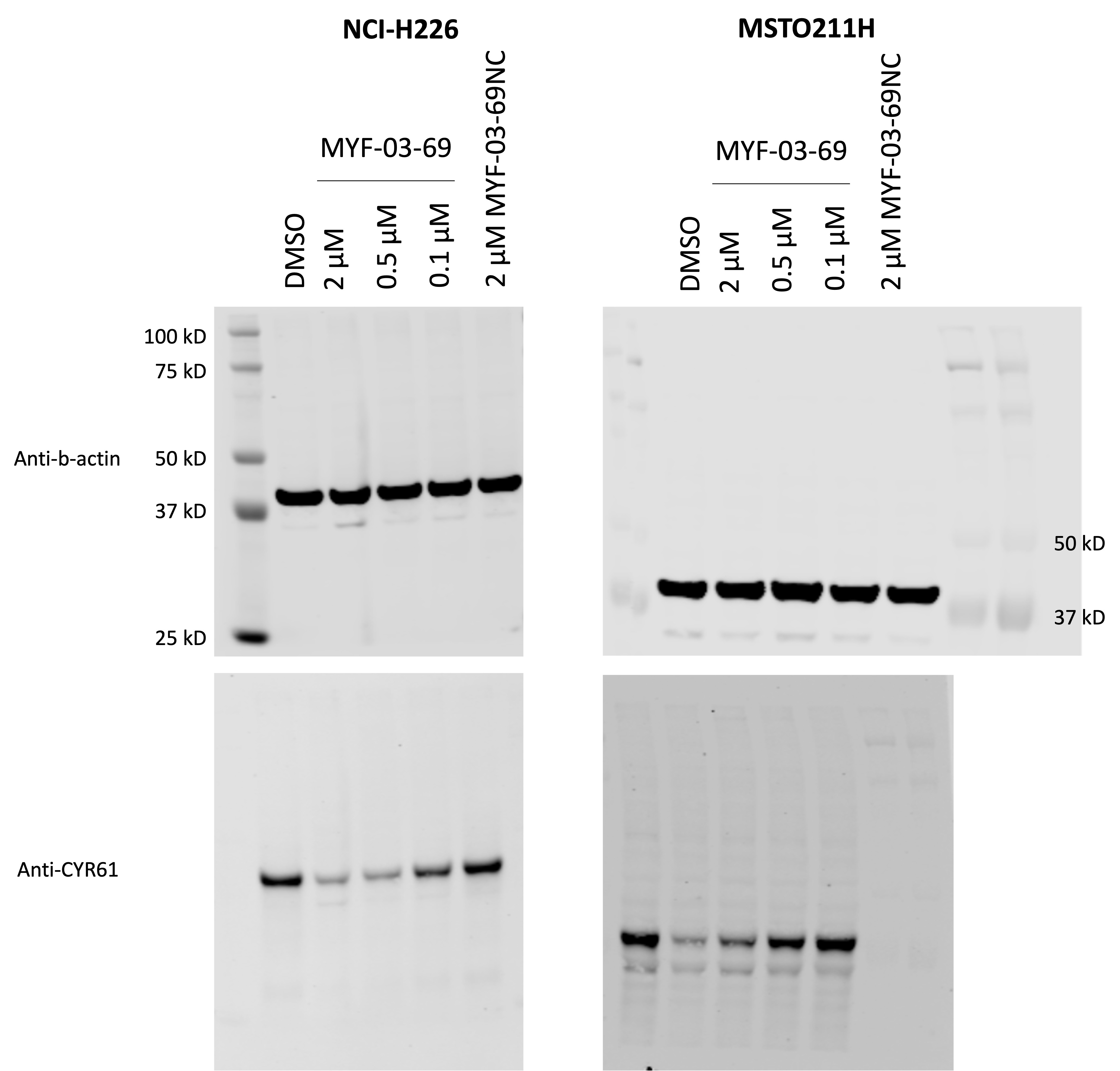

Supplement: Figure 4—source data 1. [file elife-78810-fig4-data1.zip › Figure 4-source data 2.jpg]
