# Supplementary material for: Covalent disruptor of YAP-TEAD association suppresses defective Hippo signaling
Source: eLife. 2022 Oct 27;11:e78810. doi: 10.7554/eLife.78810 (PMC9728995; doi:10.7554/eLife.78810)
Supplement: Supplementary file 1. [file elife-78810-supp1.docx]

**Supplementary Table 1 |** Table of diffraction data collection and refinement statistics for TEAD1 and MYF-03-69 co-crystal structure (deposited in the Protein Data Bank, PDB code 7LI5)

|  | TEAD1^209-424^ |
| --- | --- |
| Wavelength | 0.9792 |
| Resolution range (Å) | 35.29-1.68 (1.74-1.68) |
| Space group | P 21 21 21 |
| Cell dimensions |  |
| *a, b, c* (Å) | 36.56 89.32 135.02 |
| *α, β, γ* (˚) | 90 90 90 |
| Total reflections | 223900 (23179) |
| Unique reflections | 50567 (5044) |
| Multiplicity | 4.4 (4.6) |
| Completeness (%) | 98.24 (99.33) |
| Mean I*/*sigma(I) | 12.33 (1.24) |
| Wilson B-factor | 28.49 |
| R-merge | 0.06578 (1.381) |
| R-meas | 0.07446 (1.547) |
| R-pim | 0.03388 (0.6817) |
| CC1/2 | 0.998 (0.447) |
| CC* | 1 (0.786) |
| Reflections used in refinement | 50565 (5043) |
| Reflections used for R-free | 2571 (240) |
| R-work | 0.1939 (0.3094) |
| R-free | 0.2239 (0.3329) |
| CC (work) | 0.960 (0.725) |
| CC (free) | 0.947 (0.661) |
| Number of non-hydrogen atoms | 3761 |
| Macromolecules | 3413 |
| Ligands | 74 |
| Solvent | 274 |
| Protein residues | 424 |
| RMS (bonds) | 0.008 |
| RMS (angles) | 1.59 |
| Ramachandran favored (%) | 97.56 |
| Ramachandran allowed (%) | 2.44 |
| Ramachandran outliers (%) | 0.00 |
| Rotamer outliers (%) | 0.00 |
| Clashscore | 16.55 |
| Average B-factor | 36.89 |
| Macromolecules | 36.37 |
| Ligands | 36.40 |
| Solvent | 43.55 |
| Number of TLS groups | 20 |
|  |  |
