# Supplementary material for: Covalent disruptor of YAP-TEAD association suppresses defective Hippo signaling
Source: eLife. 2022 Oct 27;11:e78810. doi: 10.7554/eLife.78810 (PMC9728995; doi:10.7554/eLife.78810)
Supplement: Supplementary file 6. [file elife-78810-supp6.docx]

**Supplementary File 6：**

**Liver microsome stability and hepatocyte stability of MYF-03-176 and K-975**

|  | TPSA | ClogP | Mouse hepatic  microsomes t_1/2_ | Intrinsic Clearance | Mouse Hepatocyte  stability t_1/2_ | Intrinsic Clearance |
| --- | --- | --- | --- | --- | --- | --- |
| **K975** | 43.8 | 4.7 | 0.7 min | 990 μL/min/mg | 3.2 min | 433 μL/min/  1million cells |
| **MYF-03-176** | 69.5 | 3.9 | 16.2 min | 43 μL/min/mg | 22.0 min | 63 μL/min/ 1million cells |
