# Supplementary material for: Covalent disruptor of YAP-TEAD association suppresses defective Hippo signaling
Source: eLife. 2022 Oct 27;11:e78810. doi: 10.7554/eLife.78810 (PMC9728995; doi:10.7554/eLife.78810)
Supplement: Supplementary file 7. [file elife-78810-supp7.docx]

**Supplementary File 7：**

**Liver microsome stability and PK parameters of MYF-01-37, MYF-03-69 and MYF-03-176.**

|  | **MYF-01-037** | **MYF-03-69** | | | **MYF-03-176** | | |
| --- | --- | --- | --- | --- | --- | --- | --- |
| Liver microsome stability  **T_1/2_ – min** | 10 (mouse) | 25 (mouse) | | | 16 (mouse),  40 (human) | | |
| PK parameters | IV  (1 mg/kg) | IV  (1 mg/kg) | IV  (1 mg/kg) | IP  (3 mg/kg) | IV  (2 mg/kg) | IP  (5 mg/kg) | PO  (10 mg/kg) |
| **T_1/2_ – hr** | 0.33 | 0.28 | 0.28 | 0.18 | 0.84 | 0.86 | 1.6 |
| **Cl_obs_ – mL/min/kg** | 197 | 43 | 43 |  | 31 | 48 | 130 |
| **AUC_last_ – hr*ng/mL** | 84 | 425 | 425 | 880 | 1142 | 1733 | 1451 |
| **V_ss_ – L/kg** | 3.3 | 0.86 | 0.86 |  | 0.93 |  |  |
| **C_max_ – ng/mL** |  |  |  | 1100 |  | 1510 | 756 |
| **F – %** |  |  |  | 69 |  | 61 | 25 |
